# Sustained Macrophage Infiltration upon Multiple Intra-Articular Injections: An Improved Rat Model of Rheumatoid Arthritis for PET Guided Therapy Evaluation

**DOI:** 10.1155/2015/509295

**Published:** 2015-01-28

**Authors:** Durga M. S. H. Chandrupatla, Karin Weijers, Yoony Y. J. Gent, Inge de Greeuw, Adriaan A. Lammertsma, Gerrit Jansen, Conny J. van der Laken, Carla F. M. Molthoff

**Affiliations:** ^1^Department of Rheumatology, VU University Medical Center, De Boelelaan 1117, 1081 HV Amsterdam, The Netherlands; ^2^Department of Radiology & Nuclear Medicine, VU University Medical Center, De Boelelaan 1117, P.O. Box 7057, 1007 MB, 1081 HV Amsterdam, The Netherlands

## Abstract

To widen the therapeutic window for PET guided evaluation of novel anti-RA agents, modifications were made in a rat model of rheumatoid arthritis (RA). Arthritis was induced in the right knee of Wistar rats with repeated boosting to prolong articular inflammation. The contralateral knee served as control. After immunization with methylated bovine serum albumin (mBSA) in complete Freund's adjuvant and custom Bordetella pertussis antigen, one or more intra-articular (i.a.) mBSA injections were given over time in the right knee. Serum anti-mBSA antibodies, DTH response, knee thickness, motion, and synovial macrophages were analyzed and [18F]FDG(-general inflammation) and (*R*)-[11C]PK11195 (macrophages-)PET was performed followed by *ex vivo* tissue distribution. Significant anti-mBSA levels, DTH, swelling of arthritic knee, and sustained and prolonged macrophage infiltration in synovial tissue were found, especially using multiple i.a. injections. Increased [18F]FDG and (*R*)-[11C]PK11195 accumulation was demonstrated in arthritic knees as compared to contralateral knees, which was confirmed in *ex vivo* tissue distribution studies. Boosting proved advantageous for achieving a chronic model without remission. The model will offer excellent opportunities for repeated PET studies to monitor progression of disease and efficacy of novel therapeutic agents for RA in the same animal.

## 1. Introduction

Rheumatoid arthritis (RA) is an autoimmune disease that results in chronic and systemic inflammation of the joints, affecting approximately 0.5–1% of the adult population [1]. It is characterized by inflammation of the joints resulting in synovial hyperplasia by infiltration of immune cells further leading to cartilage and bone destruction [2]. Timely recognition of RA will allow for earlier start of therapy preventing more severe expansion of the disease. Moreover, several studies have shown that tight control as a treatment strategy in individual RA patients seems promising in achieving predefined level of  low disease activity or preferably remission within a reasonable period of time [3, 4]. To this end, noninvasive imaging modalities may serve as sensitive and accurate tools for assessment and monitoring of disease activity during therapy to evaluate therapeutic efficacy.

Positron emission tomography (PET) is a promising noninvasive imaging modality that can be used to visualize active arthritis at a molecular level in RA [5] via targeting macrophages [6, 7]. Most human studies targeting macrophages by PET have been performed with the macrophage tracer (*R*)-[11C]PK11195 in various inflammatory diseases [8]. (*R*)-[11C]PK11195 targets the 18-kd translocator protein (formerly known as peripheral benzodiazepine receptor) a mitochondrial membrane protein that is upregulated in activated macrophages [8]. Histological studies have shown that macrophages are an important biomarker for prediction and monitoring of therapeutic effects of a wide range of disease modifying antirheumatic drugs and biologics [9, 10]. Jahangier et al. demonstrated a clear positive clinical effect in RA patients after intra-articular treatment with Yttrium-90 and glucocorticoids correlating effect with a decrease in total numbers of macrophages [11].

Animal models can be applied for in vivo evaluation of efficacy of new therapeutic agents for RA [12]. As it takes some time for most antirheumatic drugs to read out their mode of action on arthritis activity with macrophage infiltration as a biomarker, a chronic RA animal model is required with sustained arthritis activity characterized by macrophage infiltration in synovial tissue. As currently no suitable rat model is available that would allow noninvasive macrophage PET guided evaluation of the therapeutic agents, we have optimized an antigen induced model with persistent arthritis in rats offering sufficiently sized inflamed joints to enable quantitative measurements of PET tracer uptake in inflamed joints as well as the opportunity for comparison to contralateral noninflamed control joints within the same animals.

## 2. Materials and Methods

### 2.1. Animals

Wistar rats (male, 150–200 grams, Charles River International Inc, Sulzfeld, Germany) were provided with standard food (16% protein rodent diet, Harlan Laboratories Inc., Madison, WI, USA) and water* ad libitum*. Rats were housed in groups of three or four in conventional cages and kept in a room with a 12-hour light/dark cycle and constant room temperature (21°C) and humidity level (50%). All animal experiments were carried out in accordance with the Dutch law on animal experimentation and were approved by the VU Medical Center Institutional Committee on Animal Experimentation.

### 2.2. Antigen Induced Rat Model

As reference in this paper, the methylated bovine serum albumin (mBSA) induced rat model as described by Van De Putte et al. [13] and Dijkstra et al. [14] was applied (Table 1), indicated hereafter as “original model.” In short, according to the descriptions of the original model, rats were immunized subcutaneously (s.c.) twice at days 0 and 7 with an emulsion containing mBSA (Sigma-Aldrich Chemie BV, Zwijndrecht, The Netherlands) dissolved with complete Freund's adjuvant (CFA) (Sigma Aldrich, Steinheim, Germany) and custom* Bordetella pertussis* (CBP) antigen (Becton Dickinson, Breda, The Netherlands) [14]. Rats were immunized with two administrations of 200 uL solution containing 50 mg mBSA in 1 mL 0.9% NaCl emulsified with an equal volume of complete Freund's adjuvant antigen (CFA) and custom* Bordetella pertussis* (CBP) antigen (1 × 10^11^ cells/mL). Both the first and the second immunization were performed in the tail base. At day 21, local arthritis was induced by injecting 20 *μ*L mBSA solution containing 10 mg mBSA [15] in 1 mL 0.9% NaCl intra-articular (i.a.) in the right knee (RA knee); the contralateral left knee served as an internal control (Con-RA). The i.a. injection was situated between femur and tibia and behind the patella tendon.

### 2.3. Modifications of  Original Rat Model

Three modifications were performed as compared to the original model (Table 1). At first, the second immunization (initially 200 uL in the original model) step was adapted. To minimize animal discomfort by multiple immunizations at a single location (as was performed in the original model), of second immunization was divided into two injections with one in the neck and one in the upper flank (away from the knees) with each injection consisting a volume of 100 uL. Secondly, the i.a. injection volume of mBSA was increased to 60 uL while in the original it was 20 uL. Both modifications were applied in groups A, B, C, and D.

**Figure 1:**
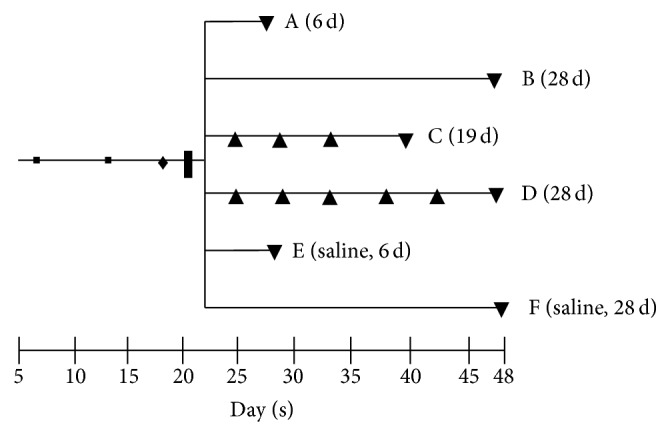
Time line of  RA rat model. 1st (■) and 2nd (■) immunization, DTH (♦), i.a. injection (*❚*), boost i.a. injections (▲), PET and/or, ex vivo tissue distribution, and (immuno-) histopathology of groups A (6 d), B (28 d), C (19 d), D (28 d), E (saline, 6 d), and F (saline, 28 d) (▼).

The last modification comprised repeated i.a. injections (resp., 3x (group C) and 5x (group D)) while in group A and B no boosts were applied. The difference between groups A and B was the sacrificing day: 6 and 28 days after i.a. injection for groups A and B, respectively (Figure 1). Control rats received an i.a. injection in the right knee with sterile physiological saline instead of mBSA. Subgroups of control rats were sacrificed at 6 (group E) and 28 (group F) days, respectively.

### 2.4. Validation Experiments

*(i) Examination of Immunization Status*

*Serum Levels of Anti-MBSA*. Blood samples before and after the immunization procedure were obtained from the tail vein with a Microvette (cb300, Sarstedt BV, Etten-Leur, The Netherlands). After centrifugation at room temperature (5 min/2500 ×g), serum samples were stored at −80°C until use. Anti-mBSA levels were determined by ELISA [16]. Briefly, 96-well microplates (Greiner bio-one, Alphen a/d Rijn, The Netherlands) were precoated overnight with 100 *μ*L/well of 5 *μ*g mBSA/mL phosphate buffered saline (PBS) at 37°C. After washing with PBS (100 *μ*L/well, 5 times) wells were blocked with 0.1% gelatin (Baker Chemical Co, Austin, Texas, USA) in PBS for 30 minutes at 37°C. Subsequently, 1 : 100, 1 : 200, and 1 : 400 diluted serum samples were added and incubated for 1 hour at room temperature. After washing with PBS, horseradish peroxidase (HRP) labeled to rabbit-anti-rat (R*α*R) IgG1 antibody 1 : 1000 (Invitrogen, NY, USA) was added to the wells and incubated for 2 hrs at room temperature. Enzyme reaction was visualized with 0.8 mg aminosalicylic acid (Sigma-Aldrich Chemie BV, Zwijndrecht, The Netherlands) and 0.5 *μ*L of 30% hydrogen peroxide (H_2_O_2_) dissolved in 1 mL distilled water. Absorption at 450 nm was measured using an ELISA reader (Tecan, Spectra Fluor, MTX Labsystems, Inc, Vienna, USA).

*Delayed Type Hypersensitivity Test (DTH)*. At day 19, immunization status was examined by DTH [17, 18] response. CBP antigen (25 *μ*L, 2.7 × 10^10^ cells/mL 0.9% NaCl) was injected s.c. in the right ear of the rat. The left ear served as an internal control. A control group of rats was injected with sterile physiological saline in the left ear. Subsequently, ear thickness was measured at 0, 6, 24, and 48 hours after injection using a digital micrometer.

*(ii) Macroscopic Evaluation of Arthritis Activity*. Macroscopic evaluation of the severity of arthritis was assessed by knee measurements prior to (at the same day) every i.a. injection. In between, knees were measured 3 times a week until rats were sacrificed. Knees were measured by caliper measurement of knee thickness in mediolateral direction.

### 2.5. Histopathology and Immunohistochemistry

Both knees were dissected* in toto* and fixed for 7 days at 4°C in 10% freshly made paraformaldehyde in PBS with 2% sucrose (pH = 7.3) prior to decalcification in 123 mM sodium ethylenediaminetetraacetic acid (Na_2_-EDTA·2H_2_O) (Merck, Darmstadt, Germany) and 113 mM sodium hydroxide (NaOH) (Sigma-Aldrich Chemie BV, Zwijndrecht, The Netherlands) (pH = 7.2) for ~5.5 weeks at 4°C. Decalcified knees were rinsed for 24 hours in 2% sucrose (Sigma-Aldrich Chemie BV, Zwijndrecht, The Netherlands) in PBS (pH = 7.2) and 24 hours in 2% sucrose in PBS and 50 mM NH_4_Cl (Sigma-Aldrich Chemie BV, Zwijndrecht, The Netherlands) (pH = 7.1). Thereafter, knees were embedded in paraffin. Sections of 5 *μ*m were cut through the center of the joint in longitudinal direction and stained with haematoxylin and eosin to assess the degree of inflammation in synovial tissue.

Immunohistochemical localization of rat macrophages was determined by a mouse anti-rat monoclonal antibody ED1 (HM3029, Hycult, PA, USA) a lysosomal membrane related antigen on rat macrophages and by a mouse anti-rat monoclonal antibody ED2 (MCBI,VU University Medical Center, Amsterdam) cell surface glycoprotein related antigen on rat macrophages [19]. An IgG1 isotype antibody (HI1016, Hycult, PA, USA) was used as negative control antibody. Briefly, after antigen retrieval with a solution of 0.1% pepsin (Sigma-Aldrich Chemie BV, Zwijndrecht, The Netherlands) with 0.1% of HCl 37% in PBS per slide for 30 min at 37°C, sections were incubated for 1 h with 1 : 100 diluted ED1, ED2, or isotype control antibody in 0.1% BSA/PBS for a period of 1 h. The detection EnVision kit (K4063 dual-link-HRP rabbit/mouse, DAKO, Glostrup, UK) was used according to instructions of the manufacturer for a period of 30 min. After washing with PBS, slides were stained for peroxidase activity with 3.3′-diaminobenzidine tetrahydrochloride (DAB) containing 0.01% H_2_O_2_. Subsequently, sections were counterstained with haematoxylin, dehydrated, and mounted. Negative controls were included by replacement of the primary antibody with an isotype specific control antibody. Images were captured using a Leica 4000B microscope and Leica digital camera DC500 (Microsystems B.V. Rijswijk, The Netherlands).

### 2.6. PET and Ex Vivo Tissue Distribution Studies

[18F]FDG with a radiochemical purity of >97% was purchased from BV Cyclotron VU (Amsterdam, The Netherlands).* (R)*-[11C]PK11195 was synthesized as described previously [20], with radiochemical purity of >98% and a mean specific activity of 95.7 ± 28.4 GBq/*μ*mol. Rats were anesthetized using inhalation anesthetics (isoflurane 2–2.5% and oxygen 0.45 volume %). The jugular vein was cannulated with a polyurethane 3 French cannula. During all procedures vital body signs like, body temperature, heartbeat, respiratory rate, and blood oxygen saturation were monitored continuously using a rectal temperature probe and pulse oxygen meter with SpO_2_ sensor. Anesthetized rats were positioned in a double-layer LSO high resolution research tomograph (HRRT) (Siemens/CTI, Knoxville, TN, USA), a small animal, and human brain 3D scanner with high spatial resolution (2.3–3.4 mm full width at half maximum) and high sensitivity [21]. First, a 6-minute transmission scan was acquired using a 740 MBq ^137^Cs rotating point source. Next, [18F]FDG (21.1 ± 5.1 MBq) or* (R)*-[11C]PK11195 (10.5 ± 2.9 MBq) was administered i.v. through the cannula and a dynamic emission scan of 1 hour was acquired. PET data were normalized and corrected for scatter, random, attenuation, decay, and dead time. Data were acquired in 64-bit list mode and converted into 16 sinograms with frame durations increasing from 15 up to 300 seconds. Images were reconstructed using an iterative 3D ordinary Poisson ordered-subsets expectation-maximization (OSEM) algorithm with 8 iterations and 16 subsets and a matrix size of 256 × 256 × 207, resulting in a cubic voxel size of 1.21 × 1.21 × 1.21 mm^3^. PET images were made of rats from the original model and of rats in group A. Sixty minutes after PET scanning, rats were sacrificed and knees, blood, and various tissues were excised and weighed and the amount of radioactivity was determined using an LKB 1282 Compugamma CS gamma counter (LKB, Wallac, Turku, Finland). Rats without PET scanning (groups B–E) were sacrificed 60 minutes after tracer injection followed by ex vivo tissue biodistribution. Results were expressed as percentage of the injected dose per gram tissue (%ID/g). PET images were analyzed using AMIDE software (Amide's Medical Image Data Examiner, version 0.9.2) [22]. Fixed size ellipsoidal shaped regions of interest (ROI) (dimensions: 6.0 × 17.7 × 7.4 mm^3^) were manually drawn over the area of the left and right knees in the last frame of the image. ROIs were projected onto the dynamic image sequence, and time-activity curve (TAC) data were extracted. TACs were expressed as standardized uptake values (SUV): mean ROI radioactivity concentration normalized for injected dose and body weight [7].

### 2.7. Statistical Analysis

Statistical tests were performed using IBM SPSS version 20. A one-sample Kolmogorov-Smirnov test was to test for normal distribution taking (*P* value) criteria for *t*-test. A Wilcoxon signed rank (exact) test was used to determine differences between paired observations (e.g., tracer uptake in right versus Con-RA knee, thickness of the antigen-induced versus control ear, and mediolateral thickness of RA knee versus Con-RA knee). A Mann-Whitney (exact) test was used to determine differences in absorbance before and after immunization. A *P* value < 0.05 was considered statistically significant. A Bonferroni correction was applied when necessary.

## 3. Results

During the entire study, no major change in body weight was observed and knee functionality was never dramatically impaired during the course of the induction of arthritis in the RA knee of the rats.

### 3.1. Immunization Status

**Figure 2:**
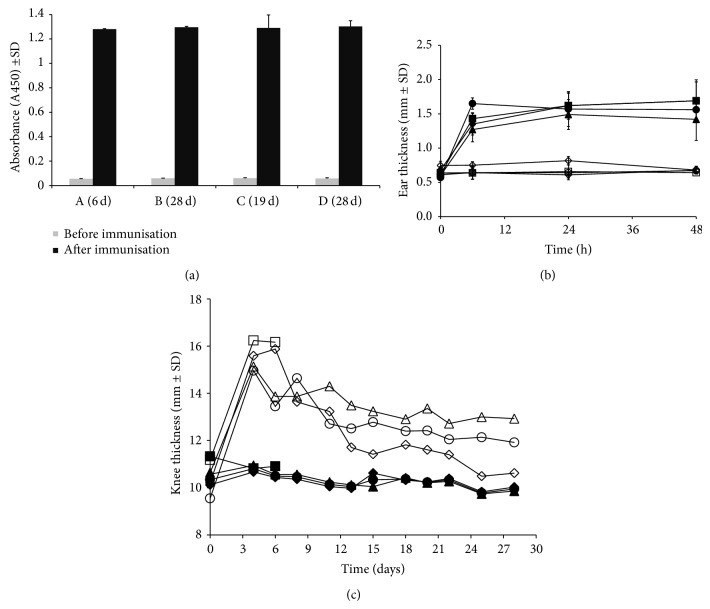
(a) Measurement of anti-mBSA in serum in rats before immunization (left) and after immunization (right) (*P* < 0.001). (b) Caliper measurement of right ear swelling of (■) A (6 d); (♦) B (28 d); (●) C (19 d); (▲) D (28 d), compared to the control ear of (□) A (6 d); (◊) B (28 d); (○) C (19 d); (∆) D (28 d), as a response to s.c. injection of antigen (*P* < 0.001). (c). Knee thickness of arthritic knee of (□) A (6 d); (◊) B (28 d); (○) C (19 d); (∆) D (28 d), compared to control Con-RA knee of (■) A (6 d); (♦) B (28 d); (●) C (19 d); (▲) D (28 d). All results depicted represent mean ± SD.

All rats showed a significant increase (*P* < 0.001) in the level of mBSA antibody titers as compared with mBSA levels before immunization (Figure 2(a)).

In addition, a DTH test was executed and all rats showed a good DTH response with a significant (*P* = 0.001) increase in ear thickness of the right ear at 6, 24, and 48 hours after injection compared with the control left ear (Figure 2(b)) and compared to control rat ear's injected with saline (data not shown).

### 3.2. Arthritis Evaluation of No-Boost Model

**Figure 3:**
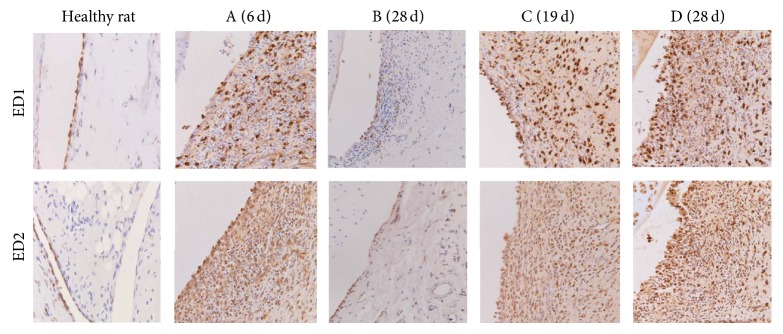
Immunohistochemistry. Images (200x) of ED1 (upper panel) and ED2 (lower panel) staining in knees of healthy rats (left panel; limited amounts of macrophages); knees from rats in the no boost groups (A (6 d) and B (28 d)); showing clear influx of positively stained ED1 and ED2 macrophages in the inflamed synovium; knees from rats in the boost groups C (19 d) and D (28 d); showing strong influx of positively stained ED1 and ED2 macrophages in the multilayered synovial linings.

As negative control, healthy rat knee sections, stained with the ED1 and ED2 rat macrophage specific antibodies, showed no signs of inflammation in the synovial tissue (Figure 3, left panels). Some macrophages were found in the single layered synovial lining. In contrast, the RA knees of the rats in the firstly adapted no-boost group (group A, 0x boost panels) showed a moderate influx of inflammatory cells in the synovium with a hyperplasia of synovial tissue consisting of 3-4 layers.

PET tracer [18F]FDG uptake in the RA knees of rats from group A was also low and showed no obvious difference as compared to the Con-RA knees, as shown in Figure 5(a).

### 3.3. Arthritis Evaluation of the Modifications in the Rat Model

#### 3.3.1. Macroscopic Evaluation of Arthritis Activity

In the no-boost group A, a significant difference was observed in knee thickness between the RA knees compared to the Con-RA knees (*P* = 0.01), which persisted until day 6 after i.a. injection (*P* = 0.01) (Figure 2(c)). In the no-boost group followed for 28 days (group B, Figure 2(c)), differences between the RA versus Con-RA knee were significant (*P* = 0.001) at day 4 but gradually decreased (*P* = 0.1) in 28 days. Continued observation without boosting demonstrated that the RA knee thickness decreased significantly (*P* < 0.0001) between 6 and 28 days (group A versus group B). Although significantly decreased in size, the knee thickness did not normalize completely at 28 days when compared to the Con-RA knees of the same rats.

When the rats were dosed with 3 boost injections (group C) significant differences were found in knee thickness of the RA versus Con-RA knee (*P* = 0.002 and *P* = 0.003 at day 4 as well as at the sacrificing day, resp.). In the group of rats dosed with 5 boost injections (group D), also significant differences were observed between the RA and Con-RA knees (*P* = 0.0001 and *P* < 0.0001 at days 4 and 28, resp.).

Comparing the boost groups C and group D (3x and 5x boosts) on the day those rats were sacrificed, a significant difference in RA knee thickness was observed, with group C < group D (*P* = 0.024). Comparing group A (no boost) with both boost groups (C and D) with respect to RA knee measurements, significant differences were observed (group A versus group C and group A versus group D: *P* = 0.003 and *P* < 0.0001, resp.).

#### 3.3.2. Immunohistochemistry

As shown in Figure 3, synovial tissue of healthy rat knees showed no signs of inflammation (Figure 3, left panel) and RA knees in group A (no boost) showed a moderate influx of inflammatory cells in the synovium with a hyperplasia of synovial tissue consisting of 3-4 layers. In contrast, the representative images of the knees from rats in group C and group D demonstrated a clear increase in ED1 and ED2 positively stained macrophages and multilayered synovial tissue (>5; see also next paragraph).

**Figure 4:**
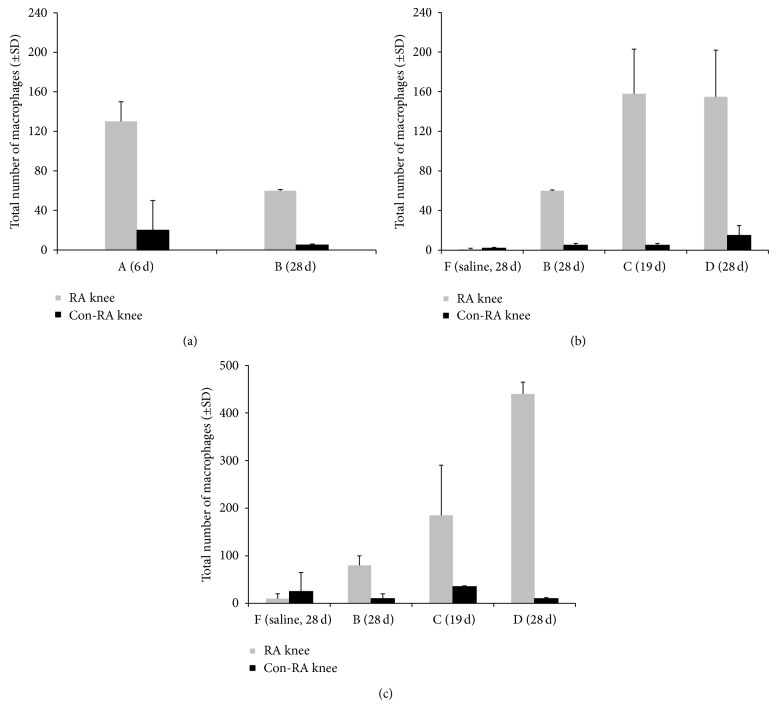
Macrophage counting in histological knee sections. (A) Total number (±SD) of ED1 positive macrophages in the lining and sublining of the knee synovial tissue from the no boost rats (A (6 d) and B (28 d)); RA knee, light grey bars, Con-RA knee, black bars. (B) Total number (±SD) of ED1 positive macrophages in the lining and sublining of the knee synovial tissue from the groups B (28 d), C (19 d), and D (28 d) are added next to saline control rats group F (28 d). (C (19 d)) Total number (±SD) of ED2 positive macrophages in the lining and sublining of the knee synovial tissue from the groups B (28 d), C (19 d), and D (28 d) are added next to saline control rats group F (28 d).

The infiltration of macrophages was quantified in the different groups of rats (between 2 and 4 rats per group). In Figure 4(a), the results for ED1 positive macrophages in group A and group B are shown. In comparison to group A, group B showed significantly lower numbers of  ED1+ macrophages (*P* = 0.028) (but still significantly higher than the control group F (*P* < 0.0001)). In Figure 4(b) (ED1) and Figure 4(c) (ED2), the ED1 and ED2 positive macrophages in knees of rats in all groups are represented, including rats injected with saline (showing very low numbers of macrophages). On comparing those numbers in group C and group B, RA knees from rats in group C clearly displayed more ED1 and ED2 positive macrophages. For those in group D an even higher amount of ED2+ macrophages was found as compared to the no-boost groups (Figure 4(c)). In Figure 3, representative images of ED1 and ED2 staining sections of different groups are shown.

**Figure 5:**
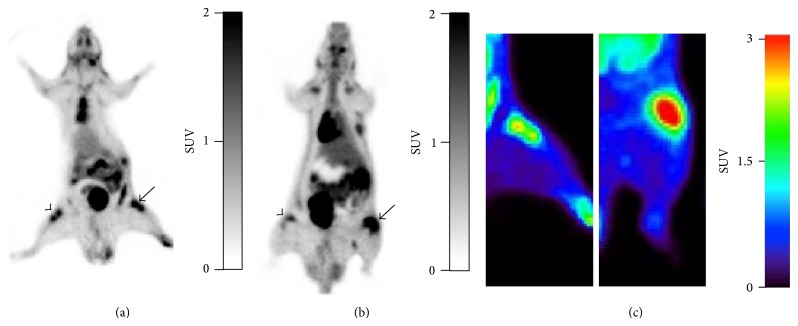
Representative coronal PET images of (a) [18F]FDG and (b)* (R)*-[11C]PK11195 in arthritic rats group A (6 d). Uptake of both tracers is clearly shown in the right arthritic knee (arrow) compared with the contralateral knee (arrowhead). (c) PET images of [18F]FDG uptake in arthritic rats, original model (left) and group A (6 d) (right).

**Figure 6:**
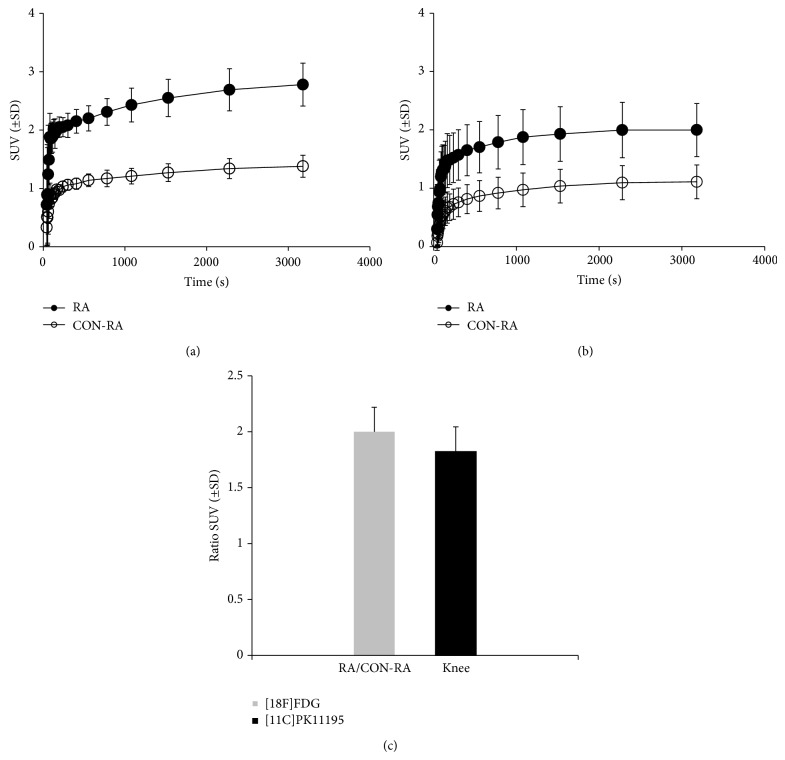
Time-activity-curves of tracer uptake expressed as SUV (± SD) in RA knee (•) and Con-RA knee (○) of (a) [18F]FDG and (b)* (R)*-[11C]PK11195 in rats of group A (6 d). (c) SUV ratios for RA/Con-RA are depicted.

*PET and Ex Vivo Tissue Distribution Studies*. The feasibility of PET evaluation of arthritis activity in the rats of group A (0x boost) as compared to the original model was assessed. Figures 5(a) and 5(b) show representative [18F]FDG and* (R)-*[11C]PK11195 images. The uptake of [18F]FDG in the RA knee of the group A was clearly higher than that in the original model (Figure 5(c), left; original model). Time-activity curves (SUV versus time after tracer injection) of the tracers up to 6 days are depicted in Figure 6. The SUV in the RA knee as compared to the Con-RA knee for [18F]FDG in group A rats was significantly increased (2.78 ± 0.366 versus 1.38 ± 0.189; *P* = 0.001; Figure 6(a)) and the ratio of uptake in the RA knee was 2 times higher than the Con-RA knee (Figure 6(c)). The SUV for* (R)-*[11C]PK11195 (Figure 6(b)) also increased in the RA knee (2.00 ± 0.55 versus 1.11 ± 0.45) and the ratio in the RA knee uptake is 1.8 times more than the Con-RA knee in group A (Figure 6(c)).

**Figure 7:**
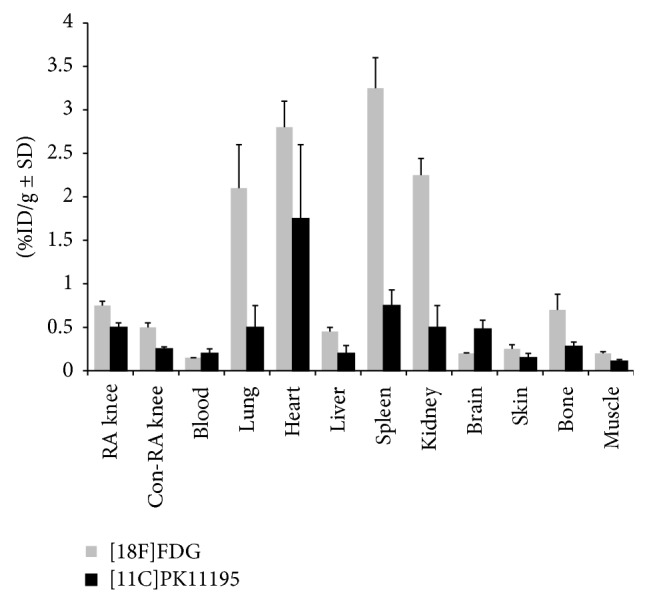
Ex vivo tissue distribution of [18F]FDG (*n* = 6), light grey bars, and (*R*)-[11C]PK11195 (*n* = 5, black bars) at 1 h after injection in rats of group A (6 d). Results are expressed as percentage of the injected dose per gram (%ID/g ± SD).

After PET, ex vivo tissue distribution of group A rats was performed and both [18F]FDG and* (R)-*[11C]PK11195 (Figure 7) showed increased accumulation in the RA knee (1.6 and 1.4 times higher, resp.) compared to uptake in Con-RA knee [18F]FDG and* (R)-*[11C]PK11195 (0.52 ± 0.06 and 0.79 ± 0.05 in RA knees; 0.33 ± 0.04 and 0.57 ± 0.05, resp.). Furthermore, both tracers showed increased uptake in macrophage rich tissues such as spleen, liver, and bone (marrow). Moreover, increased uptake of* (R)-*[11C] PK11195 in the heart could be related to TSPO expression on myocardial cells. Both tracers showed accumulation in the intestinal system and renal system (Figure 7) due to excretion of the tracers via hepatobiliary and renal route. Physiological uptake of [18F]FDG was noted in heart and brain tissue.

**Figure 8:**
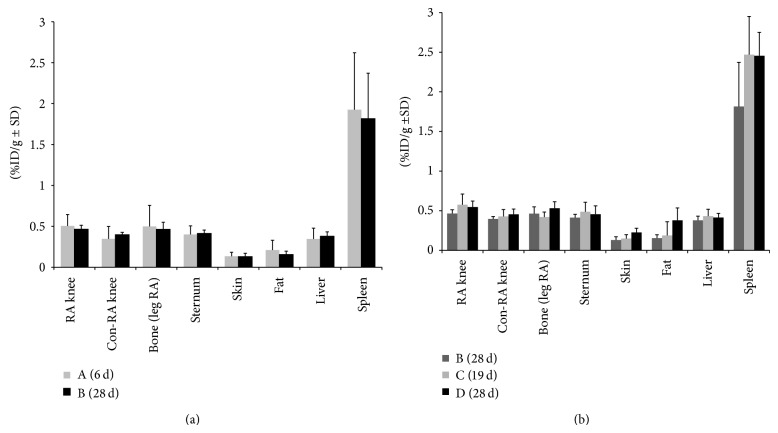
Ex vivo tissue distribution of* (R)*-[11C] PK11195 at 1 h after injection in rats of (**a**) no boost groups A (6 d) and B (28 d); (**b**) no boost group B (28 d), boost groups C (19 d), and D (28 d). Results are expressed as percentage of the injected dose per gram (%ID/g ± SD).

Results from the ex vivo tissue distribution one hour after injection of* (R)-*[11C] PK11195 in the no-boost groups (A and B) and the boost groups C and D are depicted in Figure 8. For all groups, extra-articular tracer uptake was again observed in macrophage rich tissue and physiological uptake in the intestines.

No-boost groups (A and B) (Figure 8(a)): a 1.45 times higher uptake of* (R)-*[11C] PK11195 was measured in group A between the RA and Con-RA knee. In group B, a 1.2 times higher uptake in the RA knee (0.46 ± 0.05) was found compared to Con-RA knee (0.39 ± 0.03) (*P* = 0.05). Comparing group A with B, it was observed that the tracer uptake in the RA knee from rats in group B was a little bit less (although not significantly different from that of group A).

Multiple boost group C versus group D (Figure 8(b)): the uptake of* (R)-*[11C]PK11195 in RA knees was higher in rats from group C and group D as compared to that in group B, although a level of significance was not reached between the no-boost B and group C. However, comparing no-boost (group B) with the boost group (group D) a significant difference in tracer uptake was found (*P* = 0.01).

Zooming in on the rats sacrificed at day 28 ((groups B, D, E, and F) and for group C at day 19) after the last i.a. injection a linear correlation was found between uptake and total number of ED1 positive macrophages. In contrast, no correlation could be demonstrated with respect to ED2 positive macrophages.

## 4. Discussion

In this study an mBSA-induced RA rat model was described with sustained and prolonged RA condition. Adaptations with respect to the original model described by Van De Putte et al. [13] and Dijkstra et al. [14] resulted in a model that allows evaluation of arthritis activity and therapeutic efficacy of novel antirheumatic drugs with PET. The modifications resulted in significantly higher influx of macrophages in synovial tissue, corresponding to improved visualization of arthritis with PET.

So far, no animal model was available for PET guided imaging and monitoring of therapeutic efficacy with a sustained and prolonged arthritic condition, long enough to test new drugs as well as showing some level of systemic disease. With our focus on detection of (sub)clinical arthritis versus noninflamed joints, animal models with predominant bone destruction and those with polyarticular distribution are not favored. Various rodent models of RA (acute and chronic) have been described where the very acute models are not very useful for monitoring therapeutic efficacy [23]. Chronic models such as collagen induced arthritis (CIA), bacterial (streptococcal) cell wall contents induced arthritis (SCW) and adjuvant induced arthritis (AIA) have their specific characteristics [24]. In AIA, rats develop polyarthritis with prominent bone destruction. In CIA [24] and SCW [25], swelling of paws and limbs appears with periods of remission dampening the readouts for therapeutic intervention, whereas SCW shows low significant systemic effect.

Several advantages of our present RA model are presented: it is monoarthritic; hence the contralateral knee could be used as an internal control, its robustness, and, after the immunization period, the relative rapid development of arthritis within a week characterized by clear macrophage infiltration in the synovium of one joint, resembling human RA, and with the other joints as internal control. Using the boosting procedure, the therapeutic window was largely enhanced. Macrophages play an important role in early RA and may therefore be exploited as potential targets for the development of new treatment and imaging agents [26, 27]. This model might also be particularly useful for PET purposes, since arthritis is induced in a relatively large knee joint, which is an advantage as detection of arthritis in smaller joints could be hampered by limited spatial resolution of PET scanners. Also, injection of larger volumes associated with low specific activity of some PET tracers is more problematic in mice than in rats. For monitoring response with PET imaging, quantification is essential. To this end, assessment of tracer levels in blood will be needed at any time after injection of the PET tracer which poses a problem with respect to frequent blood sampling in mice.

Limitations of the mBSA induced RA rat model are possibly the longer total time period (immunizations, i.a., and boost injections, frequent knee measurements). Also, skilled biotechnicians are needed to perform the precise intra-articular injection in the knee joint. Although the model consists of only one macroscopically inflamed knee joint, the contralateral knee also showed a low level of microscopic abnormalities related to the infiltration of macrophages in the synovium, being indicative for some systemic inflammatory effects, as also observed in a study by Meyer et al. [28]. Nevertheless, the clear difference of macrophage infiltration in the arthritic versus the noninflamed knee allowed internal comparison on PET.

PET studies showed increased uptake of both [18F]FDG and* (R)-*[11C]PK11195 in RA compared to that in Con-RA knees. Both tracers, however, have limitations for clinical imaging of arthritis activity. [18F]FDG has high sensitivity but low specificity for imaging of arthritis. In a previous clinical study, absolute uptake of RA joints and osteoarthritic joints was comparable [29]. Although* (R)-*[11C]PK11195 is a more specific tracer targeting mainly macrophages a limitation of this tracer is the background uptake in periarticular tissues [30]. The background binding of the PET tracer to both the noninflamed knee joint and periarticular bone (marrow) (in both knees and other physiological locations) could lead to underestimation of targeting properties of the applied PET tracer since PET imaging of a target relies on the contrast between the target and its background. Obtaining high, arthritic to background ratios is of particular clinical relevance to detect very early (sub)clinical synovitis as it is often only subtly present at this stage of the disease. Therefore, further research of new specific targets on activated macrophages in the early phase of RA remains warranted. An alternative candidate target in this respect is the folate receptor being selectively expressed on activated macrophages [31, 32]. Based on nanomolar binding affinities for folate and folate-conjugated ligands, this receptor might be an attractive target for both imaging and therapeutic applications, with folate linked therapeutic agents showing little or no collateral toxicity to normal tissue [33] and specifically target the activated macrophages rather than the nonactivated macrophages [34].

## 5. Conclusions

In conclusion, in this study, a rat arthritis model suitable for PET guided evaluation of antirheumatic drugs was optimized and validated. The boost regimen proved advantageous for achieving a chronic model with sustained arthritis activity. This model is excellent for in vivo testing of novel PET tracers for their suitability for imaging of arthritis (activity) as well as for PET monitoring of efficacy of novel therapeutic agents for RA.

## Figures and Tables

**Table 1:** Arthritis induction in rat: variations of the original model to the modified groups.

Code group	Original	Group A	Group B	Group C	Group D	Group E (control)	Group F (control)
Number of boost	No	No	No	3	5	No	No
Sacrificed (days post)	6	6	28	19	28	6	28

1st immunization (mBSA/CFA/CBP antigen)^*^							
No. of rats	11	3	4	3	6	4	4
Volume (*µ*L)	200	200	200	200	200	200	200
Administration route	s.c.	s.c.	s.c.	s.c.	s.c.	s.c.	s.c.
Administration location	Tail base	Tail base	Tail base	Tail base	Tail base	Tail base	Tail base

2nd immunization (mBSA/CFA/CBP antigen)^*^							
Volume (*µ*L)	200	100	100	100	100	100	100
Administration route	s.c.	s.c.	s.c.	s.c.	s.c.	s.c.	s.c.
Administration location	Tail base	Neck and upper flank	Neck and upper flank	Neck and upper flank	Neck and upper flank	Neck and upper flank	Neck and upper flank

Delayed time hypersensitivity (DTH) CBP antigen							
Volume (*µ*L)^**^		25	25	25	25	25	25
Route	s.c.	s.c.	s.c.	s.c.	s.c.	s.c.	s.c.
Administration site	Right ear	Right ear	Right ear	Right ear	Right ear	Right ear	Right ear

Local (i.a.) arthritis induction (mBSA)						Saline	
i.a. injection (mBSA^***^ or saline)	1	1	1	1	1	1	1
mBSA^#^, boost i.a. injections	0	0	0	3	5	0	0
Injected volume (*µ*L)	20	60	60	60	60	60	60
Route	i.a.	i.a.	i.a.	i.a.	i.a.	i.a.	i.a.
Administration site	knee joint	knee joint	knee joint	knee joint	knee joint	knee joint	knee joint

^*^50 mg mBSA in 1 mL 0.9% NaCl emulsified with an equal volume of complete Freund's adjuvant antigen (CFA) and custom Bordetella pertussis (CBP) antigen (1 × 10^11^ cells/mL), ^**^CBP antigen (2.7 × 10^10^ cells/mL 0.9% NaCl), ^***^10 mg mBSA in 1 mL 0.9% NaCl, and ^#^1 mg mBSA in 1 mL 0.9% NaCl.
